# Ecological landscape assessment of restored urban stream to guide adaptive management

**DOI:** 10.1016/j.heliyon.2024.e33880

**Published:** 2024-06-29

**Authors:** Jessica Tavares Machado, Gunwoo Kim

**Affiliations:** Graduate School of Urban Studies, Hanyang University, 222 Wangsimni-ro, Seongdong-gu, Seoul 04763, Republic of Korea

## Abstract

**Figure:**
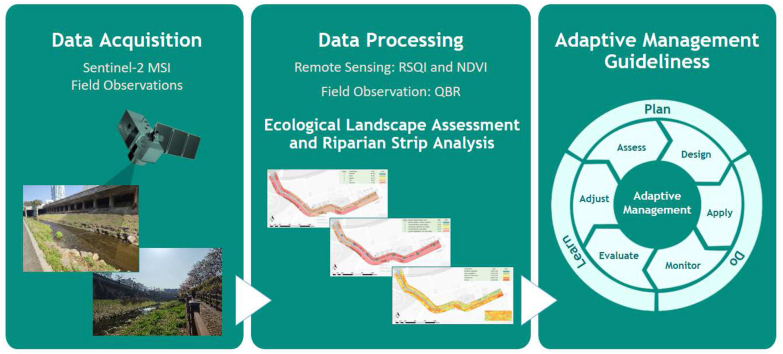

## 1 Introduction

An urban stream corridor is a vital ecological zone, providing landscape aesthetics, ecosystem services, stormwater management, and recreational opportunities. Adjacent to this corridor, the riparian forest acts as a protective buffer, effectively purifying runoff, reducing the inflow of pollutants into rivers and streams, and supporting biodiversity through habitat provision [1]. However, urban streams face more severe impacts from complex human activities compared to natural streams, being significantly damaged by changes in land use, reduction of riparian vegetation, channeling for flood control, and occupation of riverbeds [2]. While stream ecosystems with good biological integrity can typically recover quickly from natural disturbances, urbanization and the degradation of riparian vegetation have led to reduced biological integrity, altering hydrological functions, water quality, habitat availability and structure, and biotic interactions [[3], [4], [5], [6]].

In South Korea, similar to many other Asian countries, riparian strip areas have diminished, and ecosystems have been altered due to the use of floodplains for rice field cultivation or the construction of highways, resulting in the creation of degraded and unstable riverine landscapes [7]. Nonetheless, owing to the increased awareness of the benefits of nature-based solutions and in line with global trends, the Korean government has actively conducted river and stream restoration [8]. Notably, these projects predominantly emphasize partial restoration and often fail to address changes resulting from flooding disturbances, resulting in a species composition divergent from natural vegetation. Consequently, there has been a decrease in biodiversity and an increase in exotic and invasive species, a trend also observed in benthic invertebrates and fish fauna [9,10]. On the other hand, in the United States, stream restoration efforts have primarily focused on enhancing water quality and improving riparian ecosystem health to restore ecological processes and reduce nutrient loads in the water [11].

In 2010, a project was completed to restore a 2.77-km section of the Banpo Stream to its natural ecological state. However, more than a decade after the initial restoration, the stream continues to grapple with various environmental challenges, including pollution, disruptions caused by invasive species, and water odor. This outcome underscores a significant deficit in the protection and restoration of ecological conditions, as most interventions thus far have prioritized flood control and recreational activities. Consequently, excessive artificial elements have been introduced into stream corridors, compromising the quality of the natural ecosystem and, in some instances, completely eradicating native vegetation [10].

Impervious surfaces, such as driveways and artificial pavements, exert a significant impact on urban streams [12]. The increased volume and speed of stormwater runoff can result in stream channel erosion, flooding, and alterations in hydrology and water quality [13]. Urbanization-associated landscape modifications, such as deforestation and adjustments in natural drainage patterns, exacerbate these effects, contributing to nonpoint source pollution [14]. The reduction of vegetated areas and natural buffers diminishes the stream's capacity to absorb and filter runoff, leading to compromised water quality and a decline in aquatic biodiversity [2,15]. Numerous studies have evaluated changes in sediment and nutrient loads using empirical and modeling approaches. Filoso & Palmer (2011) [16] revealed that stream ecosystems exhibit relatively high nitrogen removal rates, and the uptake and retention vary with hydrological conditions, suggesting the need for restoration designs that enhance the processing and retention of nitrogen forms across diverse flow conditions. Pennino et al. (2016) [17], suggested that groundwater contamination is also a major source of urban nutrient fluxes and that combining stream restoration with the restoration of aging sewer pipes can be critical to more effectively minimizing urban nonpoint nutrient sources. Zhang et al. (2022) [18] developed indices for the environmental (potential nitrate load reduction) and economic (household willingness to pay) benefits of stream restoration. Results show higher household willingness to pay in denser urban, less wealthy neighborhoods, but lower potential nitrate load reduction. The opposite trend is observed in suburban and exurban, wealthy neighborhoods. They suggest that increased tree cover and green infrastructure investment in denser urban areas could reduce stormwater nutrient loads and enhance aquatic ecosystem health. Overall, impervious surfaces and landscape alterations have significant ramifications for the health and functionality of urban streams, underscoring the need for sustainable stormwater management practices and the implementation and conservation of green spaces to mitigate these impacts.

In this context, studies employing remote sensing offer valuable tools for comprehending the impacts of landscape alterations on urban streams. Through the utilization of satellite imagery and aerial photographs, researchers can evaluate and analyze changes in land cover, shifts in vegetation cover [19], urban expansion [20], and the prevalence of impervious surfaces surrounding urban streams [12]. Remote sensing methodologies also facilitate the estimation of various hydrological parameters, including climatic and hydrological fluctuations [21], surface runoff, streamflow, and alterations in stream channel morphology [22]. Through comparisons of remote sensing data, researchers can quantify the impacts of urbanization on urban streams, encompassing changes in stream health, water quality [23], and habitat degradation.

Several remote-sensing-based indices have been used to assess the quality and health of riparian strips. In a study by Novoa et al. (2018) [22], the Riparian Strip Quality Index (RSQI), which is based on the percentage of the riparian area and weighted classes, was used with Object-based Image Classification and the Riparian Strip Drainage Importance to develop a new indicator, the Riparian Strip Efficiency Index, characterizing the ecosystem services of riparian strips for use in agricultural areas. Saha et al. (2020) [24] combined the RSQI with the Normalized Difference Vegetation Index (NDVI) and Qualitat del Bosc de Ribera (QBR) index and conducted a user perception survey to explore the ecological and aesthetic quality of riparian areas in India. Additionally, Rivas-Fandiño et al. (2022) [25] employed the RSQI and QBR indices to monitor changes in riparian vegetation of a river that suffers from multiple anthropogenic pressures. The RSQI and QBR indices are frequently integrated because they serve complementary purposes. The RSQI evaluates riparian quality by considering area proportion, while the QBR concentrates on various aspects of vegetation, channel quality, and structure [26]. This combination of the two indices allows for a more comprehensive and well-rounded assessment. Das et al. (2023) [27] developed a remote sensing-based index to assess urban environmental efficiency (UEE) in the Kolkata Metropolitan Area (KMA) from 2000 to 2020. Their findings indicate deteriorating UEE over time, particularly in urban areas, with factors like greenness, vegetation health index (VHI), and wetness significantly impacting UEE.

While these studies have contributed valuable insights into understanding and analyzing the ecological landscape and riparian strip quality, there remains a research gap in providing evidence-based guidelines to improve urban stream restoration practices. Therefore, this study aims to address this gap by proposing guidelines using the adaptive management approach to enhance the effectiveness of restoration efforts beyond landscape analysis.

To improve the efficiency of urban stream restorations, numerous practitioners are adopting the adaptive management approach [28]. This method enables them to continually monitor and evaluate the outcomes of their restoration efforts, facilitating adjustments and refinements based on observed results [8]. Adaptive management involves setting clear restoration goals, implementing restoration actions, and actively monitoring various indicators such as water quality, habitat conditions, and biological communities within the stream ecosystem [28]. This dynamic and responsive approach allows for the incorporation of new information, scientific advancements, and stakeholder inputs throughout the restoration process [29]. However, one challenge in implementing adaptive management includes the lack of suitable guidelines for assessing river restoration project outcomes, which are essential for adaptive management, project efficiency evaluation, future program optimization, and public acceptance [30].

Thus, by conducting remote sensing-based landscape pattern analysis and riparian vegetation evaluation, this research seeks to contribute valuable insights to urban stream management providing guidelines for adaptive management to promote healthy and sustainable urban stream environments. These findings may provide evidence-based recommendations for landscape and urban planning professionals working in urban water management, emphasizing the potential role of healthy ecosystem services, such as regulating, supporting, provisioning, and promoting cultural value. Ultimately, this study strives to improve understanding of strategies that can positively impact Banpo Stream's ecological quality and derive implications for the sustainable use of urban streams.

## 2 Materials and methods

### 2.1 Study area

**Fig. 1:**
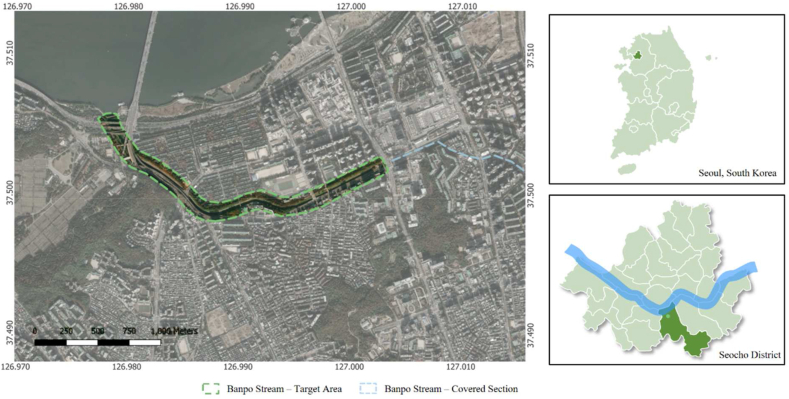
Location of the banpo stream and target area within Seoul and South Korea.

The spatial scope of this research focuses on the Banpo Stream in the Seocho District, Seoul City, South Korea (Fig. 1).

The Banpo Stream (37°30′06″N to 37°30′19″N, 127°00′06″E to 126°58′42″E) is a local stream with a length of 4.86 km (2.09 km covered) and a basin area of 27.87 km^2^. The stream is the first tributary of the Han River, one of the longest rivers in the Korean Peninsula. It originates from Umyeon Mountain, flows northwest from a relatively large area, merges with Sadang Stream, which is a fully covered tributary, and finally flows into the Han River. It is a flat riverbed area with a low elevation and silt loam soil. The landscape analysis was carried out on a 100-m buffer on each side of the stream, comprising an area of 0.527 km^2^.

### 2.2 Field survey, image acquisition, and pre-processing

**Fig. 2:**
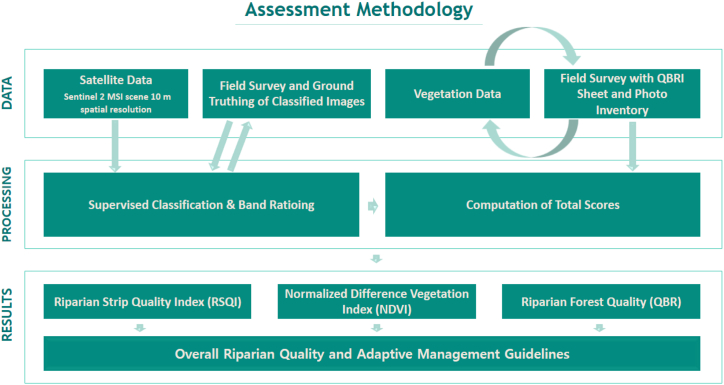
Methodological flowchart of the study highlighting the data acquisition, processing and results.

Field observations and mapping were conducted to analyze the landscape characteristics and riparian vegetation quality. A methodological flowchart is presented in Fig. 2 to facilitate the comprehension of the assessment methodology.

The riparian area was analyzed using the RSQI and NDVI as measures. The RSQI index is based on Land Use Land Cover analysis; for the calculation, this study used aerial photographs and one Sentinel 2 MSI scene with 10 m spatial resolution imaged on January 12, 2023, with a low presence of clouds (−10 %) for better quality results. The same images were used to calculate the NDVI, and the process was conducted using ArcGIS 10.8 for both indices. Field surveys were conducted from April 2022 to January 2023, before and after the mapping analysis, to ground truth the image-extracted information and calibrate the mapping indices using the QBR index.

**Fig. 3:**
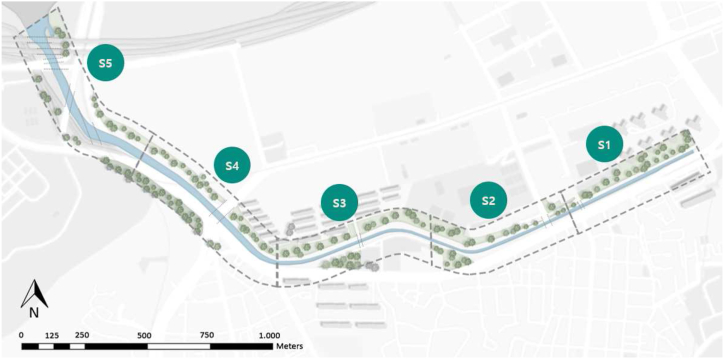
Division of the study area into five sections based on the configuration of the channel and the riparian strip vegetation.

The site was divided into five sections based on discerned changes in the stream and riparian vegetation profiles from adjacent sites (Fig. 3).

Each section was approximately 500 m long and enumerated from the entrance to the east side.

### 2.3 Riparian strip quality index

The RSQI is a measure of the ecological quality of riparian strips computed using weighted riparian land-cover classes [31]. Using the RSQI, researchers and environmental managers can monitor changes in the riparian zone over time, compare different sites or regions, and identify areas that may require restoration or conservation efforts. Each class was assigned a weight according to Saint-Jacques and Richard (1998) [31] with minor adjustments: tree cover 10, grassland 5.8, bare soil 1.7, water body 3.8, built-up 1.9, and infrastructure 1.9. The index is calculated as follows (Eq. (1)):

(1) $$RSQI=\frac{\sum \left(\%{LU}_{i\times }W_{i}\right)}{10}$$

where *%LUi* represents the land cover percentage, *Wi* is the weight of the *i*th land use class in the riparian buffer, and 10 is the highest possible score. For this study, a length of approximately 540 m and a buffer of 100 m on each side of the stream was defined for the analysis of the riparian strip, as this scale contains great landscape variation and is often used in the biological assessment of some quality elements of small streams [32]. The area of the classes was calculated separately for each stream bank per section, and then the mean value was calculated and used to classify the quality of the riparian vegetation as follows: Excellent (90–100); Good (75–89); Average (60–74); Low (40–59), and Very Low (17–39).

### 2.4 Normalized Difference Vegetation Index

The NDVI is widely employed to assess the density and health of vegetation in a given area, making it one of the most frequently used vegetation indices. The range of the NDVI extends from −1 to +1, with higher NDVI values indicating dense and flourishing vegetation. Conversely, lower or negative NDVI values are typically observed in water bodies, bare rocks, and human-made structures [33]. Therefore, it is also helpful to analyze landscape quality based on land cover and vegetation robustness, calculated as follows (Eq. (2)):

(2) $$NDVI=\frac{NIR-RED}{NIR+RED}$$

where NIR is the spectral reflectance measurement in the near-infrared areas and RED in the red areas. To evaluate the NDVI combined with the QBR and RSQI parameters, the calculated values were converted to a scale of 1–5 based on the characteristics of the vegetation and soil cover classes identified in the study area.

### 2.5 Qualitat del Bosc de Ribera index

The QBR index (in English, riparian strip quality index) is independent of other stream features and is relatively simple, allowing the easy evaluation of various essential factors of riparian vegetation, including its extent and arrangement, aspects of the riparian zone's physical characteristics, and human impacts on the landscape [26].

The index is divided into four sub-indices: total riparian cover, cover structure, cover quality, and channel alteration. Each of these sub-indices were initially assigned one of four values: 0, 5, 10, or 25, without the possibility of intermediate values. Deductions from the initial value occur when metal roads, non-native species, human-made features along the river, or structures obstructing the main channel are present. The final QBR score was obtained by summing these four subindices, and if the final value was negative, it had to be converted to zero, because the scores varies between 0 and 100. According to the QBR index, five quality classes of riparian habitat are suggested: ≥95: Riparian habitat in natural condition; 75–90: Some disturbance, good quality; 55–70: Disturbance important, fair quality; 30–50: Strong alteration, poor quality, and ≤25: Extreme degradation, bad quality [26].

In this study, the index was calculated based on the observations of eight stations along the Banpo Stream on three dates: April 3, 2022; February 3, 2023, and February 5, 2023, using the field sheet and guidelines provided by the University of Barcelona [26]. All observations were carried out individually by the author, and the final scores were assigned after local identification of vegetation species and analysis (native, non-native, invasive species) in the laboratory.

## 3 Results

### 3.1 Riparian strip quality index

**Fig. 4:**
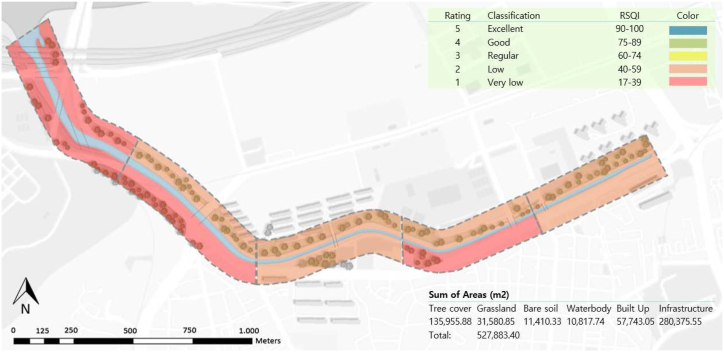
RSQI Final Result Map indicating the rating calculated per section analyzed. The rates indicate low (scores between 40 and 59, rate 2) and very low (scores between 17 and 39, rate 1) riparian quality.

Using ArcGIS software and the maximum likelihood classification method, six classes were identified in the 100 m buffer from the stream margins: tree cover, grassland, built-up area, bare soil, water body, and infrastructure (roads and bridges). After the classes were identified, aerial photographs from Naver and Google Maps were used for comparison and accuracy analysis. The user, Producer and Cohen's kappa metrics [25] were calculated using 50 randomly selected points to verify the mapping accuracy, resulting in an overall accuracy of 92 % and Cohen's kappa of 89 %. The weighted riparian land cover class method was used to calculate the RSQI. The values were calculated separately for each of the five sections and sides of the stream buffer for detailed visualization, and the final scores (Table 1, Fig. 4) were calculated by combining both sides.

**Table 1:** RSQI score results.

Right Riparian Strip								
Sector	Tree cover (W = 10)	Grassland (W = 5.8)	Built Up (W = 1.9)	Bare Soil (W = 1.7)	Waterbody (W = 3.8)	Infra (W = 1.9)	Total Area	RSQI
S1	42	10	12	0	0	35	100	57
S2	29.7	7.2	13.1	1.4	2.5	46.2	100	46
S3	28.66	9.57	8.59	4.4	0	48.78	100	46
S4	21.8	8.7	11.5	7.1	2.1	48.6	100	40
S5	18.9	8.8	8.6	2.2	4.2	57.3	100	38
Mean								45
**Left Riparian Strip**								
S1	30.46	0.06	9.97	0.44	0	59.06	100	44
S2	22.6	3.1	12.9	0.1	2.1	59.3	100	39
S3	37.53	5.41	12.7	4.39	0	39.98	100	51
S4	12.6	2.3	12.8	0.3	1.1	70.8	100	30
S5	16.8	5.1	8	1.6	6.6	61.9	100	36
Mean								40
**Combined**								
S1	37	5	11	0	0	47	100	**51**
S2	26.48	5.41	13.28	0.76	0	54.08	100	**43**
S3	33	7.54	10.6	4.39	0	44.47	100	**49**
S4	17.19	5.63	12.09	3.67	1.58	59.83	100	**35**
S5	17.69	6.24	9.09	1.36	2.16	63.45	100	**36**
Mean								**43**

Out of a total area of approximately 527,833 m^2^, over half (280,375 m^2^, 53.12 %) comprises infrastructure, including highways, bridges, and sidewalks. The tree cover class occupies the second largest area (135,955 m^2^, 25.75 %), which is less than half the size of the infrastructure area, despite its crucial role in maintaining the quality of the riparian strip. The remaining classes were arranged in descending order, with built-up areas (57,743 m^2^, 10.94 %), followed by grassland (31,580 m^2^, 5.98 %), bare soil (11,410 m^2^, 2.16 %), and water bodies (10,817 m^2^, 2.05 %).

With scores ranging from 0 to 100, the riparian strip quality can be classified from 1 to 5, where 1 indicates very low quality and 5 indicates excellent quality [22]. Because of the Dongjak subway station and the elevated road structures in section five, this area received the lowest score. The areas close to the apartment complexes (sections 1, 2, 3, 4) had a larger vegetation area and therefore, had a better score, but were still classified as low quality.

### 3.2 Normalized Difference Vegetation Index

**Fig. 5:**
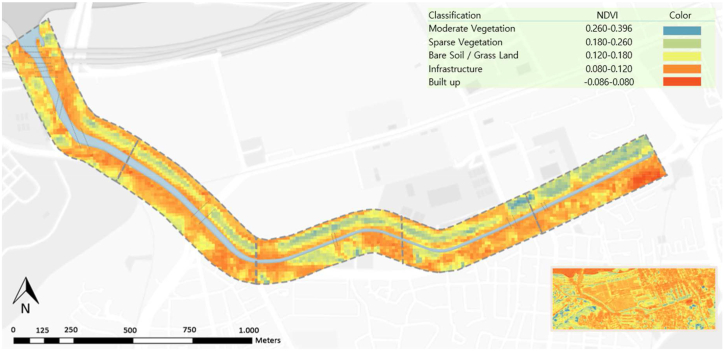
NDVI Final Result Map indicating the rating calculated per vegetation robustness/land cover. The values ranging between −0.086 and 0.396 indicate that most of the area is composed of non-vegetated areas, with higher values indicating sparse vegetation in the strip near the apartment complex.

The NDVI was calculated with values ranging between −0.086 and 0.396 (Table 2, Fig. 5), a relatively low span considering that healthy vegetation in general has values from 0.8 to 0.9 [34]. The results showed that areas with denser forest cover consistently exhibited NDVI values above 0.26, corresponding mostly to the landscaped vegetation in the stream promenade and near apartment complexes. By contrast, bare soil, grassland, and sparse vegetation areas displayed lower NDVI values ranging from 0.12 to 0.26. This suggests that these regions have lower vegetation densities because of land-use practices and urban development. Furthermore, the analysis identified most areas with extremely low NDVI values, below 0.12, indicating the presence of water bodies or other non-vegetated surfaces, including primarily paved surfaces. To evaluate the NDVI combined with the QBR and RSQI parameters, the calculated values were converted to a scale of 1–5 based on the characteristics of the vegetation and soil cover, being: Water and built-up areas −0.086–0.080 = 1; Infrastructure 0.080–0.120 = 2; Bare soil/Grassland 0.120–0.180 = 3; Sparse Vegetation 0.180–0.260 = 4, and Moderate Vegetation 0.260–0.396 = 5.

**Table 2:** NDVI score results.

Section	Minimum	Maximum	Mean	Rating
Section 1	−0.075	0.370	0.118	2
Section 2	−0.052	0.367	0.091	2
Section 3	−0.020	0.319	0.092	2
Section 4	−0.048	0.262	0.069	1
Section 5	**−0.086**	**0.396**	0.073	1
**Total area**	−0.0562	0.342	0.089

#### 3.2.1 Qualitat del bosc de ribera

In the case of the QBR, unlike the RSQI, both stream margins were considered as a single unit for all conditions. The field surveys were carried out on three different days (April 3, 2022; February 3, 2023, and February 5, 2023), and eight observation stations were identified based on the presence of elements that would significantly influence the final scores. The scores for each section for each evaluation category are presented in Table 3, and the details of field observation notes are presented in Table 4.

**Table 3:** QBR score results.

Section	Station	TRC Score	CS Score	CQ Score	CA Score	Total QBR Score	Mean (17)	QBR Category
Section 1	1	0	0	10	0	10	10	Very bad
Section 2	2	0	0	10	0	10	15	Very bad
	3	0	10	10	5	25		
Section 3	4	0	0	20	0	20	20	Very bad
	5	0	0	20	0	20		
Section 4	6	0	0	0	0	0	10	Very bad
	7	0	0	15	0	15		
Section 5	8	0	10	20	0	30	30	Bad

TRC: Total Riparian Cover. CS: Cover Structure. CQ: Cover Quality. CA: Channel Alteration.

**Table 4:**
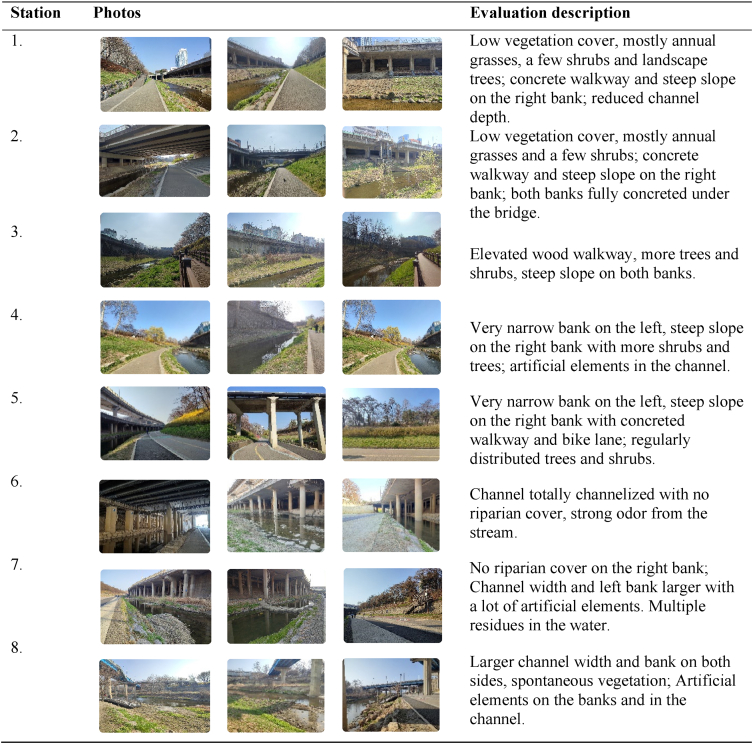
Field observations notes for calculating the QBR Index.

**Fig. 6:**
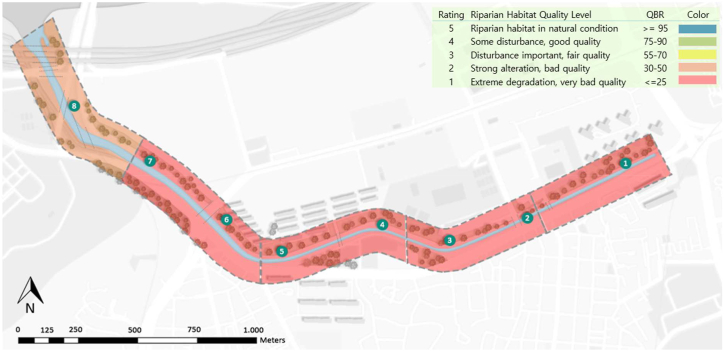
QBR Final Result Map indicating the rating calculated per section analyzed along the eight observation points. Sections 1, 2, 3, 4 were rated as very bad quality with extreme degradation of the riparian strip (scores equal or below 25), while section 5 was rated as an area with bad quality of the riparian strip due to strong alteration (scores between 30 and 50).

Although the stream channel is narrow, there are some sections with a reasonable number of shrubs and trees; however, the disconnection due to the concrete path and steep slope of the embankment significantly reduces the final value assigned. In contrast to the RSQI and NDVI indices, as illustrated in Fig. 6, section five achieved the highest score due to the expansive presence of natural vegetation along the stream and minimal human intervention. This is attributed to the inaccessibility of the left side of the stream, which resulted in the growth of spontaneous vegetation. Station six exhibited null scores across all categories due to its complete concrete coverage and the presence of a highway structure. This area requires major attention for improvement as the environment is dark and visually unpleasant. Station three in section two was the only area that scored in the channel alteration category. Although the section is modified by discontinuous rigid structures along the margins, which is equivalent to five points, there are no rigid structures such as wells on the riverbed or transverse structures in the channel, as present in the other sections; therefore, there is no deduction in the score.

A significant portion of the vegetation is composed of landscape vegetation (*Prunus serrulata, Forsythia koreana, Rhododendron yedoense, among others)* which deviates from a natural arrangement. This indicates that the vegetation has been intentionally designed, modified, or managed for specific aesthetic or functional purposes, rather than being in its original, naturally occurring state. Landscape vegetation typically consists of plants and trees that are strategically chosen and arranged to serve practical and ornamental purposes. While such landscaping practices can enhance the aesthetic appeal and functionality of urban and suburban areas, they may not always align with the ecological composition and spatial patterns found in natural environments [35]. Introducing non-native or exotic species into ecosystems can have profound ecological impacts. These species may outcompete native flora and fauna, leading to habitat fragmentation and a reduction in available wildlife habitat. Additionally, non-native species may not have natural control in their new environment, allowing them to proliferate unchecked and alter nutrient cycling dynamics. These changes can disrupt ecosystem balance and resilience, ultimately threatening the long-term sustainability of the landscape [1,10,15].

Disturbance by invasive species was also identified at the site. Sections 2, 3, 5 exhibit a heightened prevalence of invasive plant species. The species *Ambrosia trifida*, *Lactuca scariola*, and *Humulus japonicus* were predominantly observed, with the latter predominantly concentrated within section 2. *Ambrosia trifida* is a highly invasive weed species globally, listed as one of the most ecologically destructive weeds. Dominating communities and decreasing plant diversity, it's classified as a severe nuisance, particularly in South Korea [36,37]. *Lactuca scariola* and *Humulus japonicus* quickly invade forest canopy gaps and open areas along rivers and streams, hindering the growth of native plants. Its rapid growth also overwhelms tree and shrub regeneration, further impacting ecosystem diversity and health [38,39]. Furthermore, invasive species can reduce water quantity and impact ecosystem functions related to water temperature, edaphic characteristics, and physical and chemical alteration in allochthonous inputs [40]. The introduction and dissemination of invasive alien species have the potential to present ecological, economic, and societal risks [41], therefore, being a concerning disturbance that needs to be addressed and managed to protect the ecosystem and biodiversity.

Accordingly, the stream area had an average value of 17 out of 100, which is a very low value, indicating that the area had extreme degradation and very poor quality.

### 3.3 Pearson's correlation analysis

Correlations between the indices were explored using Pearson's correlation analysis. The correlations of RSQI and NDVI were very high, positive, and statistically significant (r = 0.921, p < 00.05), whereas the correlations of QBR with RSQI and NDVI were low, negative, and not significant: r = −0.305, p < 0.618; and r = −0.409, p < 0.494, respectively). RSQI and NDVI are indices based on remote sensing and, therefore, do not identify elements under structures, as in the case of Section 5, where a partial area of the stream is covered by the structure of elevated roads. Likewise, while sections 1, 2, 3, 4 near the apartment complex scored better on mapping-based indices, the field observations considered for the calculation of the QBR demonstrated that the vegetation was disconnected from the stream, and several small-scale artificial elements that also accounted for score deduction were not identified by the remote-sensing imagery or represented very small pixel values.

## 4 Discussion

The Banpo Stream is a small and narrow urban stream that has had its riparian area profoundly altered by land occupation. The lack of a natural arrangement in the riparian strip can have various implications for the ecosystem. It may affect the distribution and abundance of native species, disrupt ecological interactions, alter habitat suitability for wildlife, and influence the overall ecological balance of the area [42]. Moreover, the introduction of non-native or invasive species in landscape vegetation can further impact local biodiversity and ecological processes such as competition, pollination, and nutrient cycling [[36], [37], [38], [39], [40]]. Understanding the prevalence of landscape vegetation and its departure from a natural arrangement in the study area is crucial for assessing the potential ecological consequences and identifying opportunities for restoring or enhancing ecological integrity. It also highlights the need for informed decision-making when designing and managing urban landscapes to strike a balance between human needs and ecological considerations.

Furthermore, the predominance of pervious surfaces, such as concrete embankments, driveways, bike lanes, and walkways impedes natural water infiltration, leading to increased runoff and potential flooding during storm events, carrying and accumulating pollutants in the water body [12]. The construction of concrete embankments disrupts natural stream flow dynamics, while roads built parallel to the stream cause vegetation disconnection, leading to reduced riparian vegetation area. These alterations contribute to habitat loss, fragmentation, and decreased biodiversity in the stream environment, further leading to elevated water temperatures, degraded water quality, and impaired nutrient cycling [43].

### 4.1 Guidelines for adaptive management

Considering the restoration carried out in the Banpo Stream, similar to other cases in Seoul, the adoption of adaptive management is recommended to encourage the constant reassessment of previously applied practices, learning and improvement of techniques. To develop successful adaptive management, it is necessary to recognize the complexity of stream ecosystems in the urban environment and the need for continuous learning by monitoring. Therefore, this study presents the steps and improvement points to be considered in the development of an adaptive management plan classified into two main areas: the methodology for implementing adaptive management, and the aspects to be addressed for improvement. Regarding the methodology, these recommendations align with the guidelines outlined by Levine (2004) [28] and are elaborated as follows.

#### 4.1.1 Defining problems, goals and objectives

By identifying ecological landscape issues, this study's findings serve as a reference for future stream restoration initiatives. The main challenges highlighted include the high proportion of impervious surfaces hindering water infiltration and habitat creation, and natural ecosystem disruption. Likewise, establishing well-defined objectives and transparently communicating them to all stakeholders is crucial for the effective implementation of adaptive management [28]. Involving diverse interest groups is needed for stream restoration and monitoring [44], especially in densely populated areas likeSeoul, as it can foster acceptance, benefit from diverse perspectives, gain economic support, and promote community cohesion.

#### 4.1.2 Developing a conceptual model and monitoring plan

It is key to create hypotheses about the effects of management actions, and design experiments to test these hypotheses. With the rapid advancement of technology and integration of smart city approaches into management, artificial intelligence can assist in creating predictive models to forecast outcomes [45]. The lack of comprehensive monitoring of the Banpo Stream and other urban streams at various spatial and temporal scales hinders abilities to learn from past experiences and advance restoration and management practices. Therefore, by using the monitoring results as a basis, decisions should be made, and interventions implemented.

#### 4.1.3 Evaluating results against goals and hypotheses

The learning process involves comparing results with pre-established objectives and hypothesis models. Although one of the goals of the Banpo Stream restoration project was to improve the ecological condition of the stream, this study demonstrates that the practices applied were not effective in achieving this objective. Therefore, is important to continuously reassess and readjust the problem statement, goals, conceptual model, interventions, and monitoring plan accordingly. This includes re-evaluating the problem statement, refining goals, updating the conceptual model, modifying interventions, and adjusting the monitoring plan.

Furthermore, several relevant suggestions concerning restoration practices based on the Banpo Stream assessment are identified and presented as follows.

#### 4.1.4 Landscape scale

In addition to the challenges associated with local-scale restoration practices, there are issues with the disconnection of green areas caused by roads and construction, which negatively impact habitat provision and ecological quality. In Seoul, all 35 urban streams are connected through the Han River, presenting an opportunity to integrate them into a cohesive and independent network [46]. Although this study focused only on the surroundings of the stream, assessing urban streams at different scales is crucial for a comprehensive understanding of their ecological health and function. By considering multiple scales from the local reach to the watershed level, it would be possible to develop effective strategies for the conservation, restoration, and integration of urban streams into the urban landscape. Furthermore, urban streams with riparian vegetation can act as green corridors, linking fragmented green spaces, and allowing the movement and dispersal of wildlife [47]. They also contribute to the overall green infrastructure of a city by providing ecosystem services such as stormwater management, water purification, and temperature regulation. Hence, future adaptation and restoration plans should consider the city scale to facilitate the reconnection of green spaces and maximize the benefits of healthy ecosystem services.

#### 4.1.5 Hydrological considerations

The surroundings of Banpo Stream are mostly occupied by roads and paved structures, and pollutants from highways are directly discharged into the stream during periods of heavy rainfall. Numerous studies have demonstrated the detrimental effects of pollutant discharges on aquatic habitats and water quality [12,20,48]. To mitigate this issue, incorporating principles like Blue-green Networks [49] can facilitate the implementation of integrated strategies that promote sustainable urban development accounting for hydrological conditions. Implementing such strategies as rain and bioretention gardens, green corridors, and green roofs would collectively enhance rainwater infiltration, decrease pollutant inflow into water bodies, and minimize the likelihood of flooding. Addressing upstream challenges arising from impervious surfaces in urban watersheds simultaneously is also important [50]. This can be achieved by reducing directly connected impervious areas instead of adopting partial mitigation strategies [51], and encouraging infiltration and detention storage through the implementation of diverse grey and green infrastructure solutions, such as increased tree canopy, vegetated roadside bioswales, and permeable pavement [52]. Is important to notice, however, that the application of these methods requires extensive study and analysis, to avoid adverse effects.

#### 4.1.6 Design aspects

Many restoration projects follow a generalized pattern, overlooking site-specific characteristics. Incorporating cultural elements into these projects would foster a sense of place and stronger connections among the residents [53]. Therefore, future studies should focus on location-specific features. Additionally, the application of the concept of universal design [54] is suggested. Reducing and optimizing the number of accesses and replacing the concrete sidewalk with elevated boardwalks could increase the infiltration area on the stream embankment and allow the growth of vegetation, creating a species movement corridor.

### 4.2 Ecological landscape issues

The prevalence of paved roads, parking lots, and buildings in the Banpo Stream riparian area impede water infiltration, leading to several ecological, social, and environmental issues. Reduced water infiltration limits habitat creation and exacerbates the risk of flooding, whereas the accumulation of polluted runoff from nearby roads contributes to the degradation of stream water quality [12,55]. Pollutants, such as heavy metals, oils, and sediments, negatively impact aquatic ecosystems and compromise water quality and biodiversity. Furthermore, the construction of roads around streams isolates vegetation and prevents the creation of green networks and corridors for the movement of species and the protection of biodiversity [56].

Vehicle emissions, including nitrogen oxides, and particulate matter, contribute to air pollution resulting in respiratory problems, cardiovascular issues, and heightened risks of asthma and allergies. Additionally, traffic noise from roads and highways can have negative impacts on human health, leading to stress, sleep disruptions, hypertension, and impaired cognitive function [57]. Moreover, roads and highways can physically divide communities, leading to reduced social interactions, and limited access to amenities and services, as is the case of the Banpo Stream where residents of the southern area have limited access due to the highway. On the other hand, optimizing the landscape pattern is an effective measure to reduce non-point source pollutants [14] and reintegrate disconnected areas. Moreover, in the context of urban stream restoration projects, it's crucial to recognize the interconnectedness between vehicle emissions, traffic noise, community fragmentation, and the health of riparian ecosystems. These factors directly impact the stream environment and surrounding communities in areas like the Banpo Stream, which is situated amidst urban development and transportation infrastructure. Integrating natural resources and optimizing landscape patterns in restoration efforts can effectively mitigate air and noise pollution, promote social cohesion, and enhance overall ecosystem resilience [58].

This study also identified shortcomings in stream and urban river restoration practices. The emphasis on recreational activities and landscape aesthetics over ecological quality, coupled with the urbanization-induced fragmentation of green areas, presents significant challenges in reconnecting these spaces. Accordingly, policymakers and urban, landscape and ecology practitioners should partner to find sustainable alternatives to reconnect the city's streams and river corridors.

### 4.3 Limitations

The integration of remote-sensing-based indices to analyze land cover with an index based on field observations was essential to provide a comprehensive analysis of the stream ecological conditions. Complementary on-site analyzes using the QBR index helped demonstrate that even in vegetated areas identified on mapping analysis, the quality, configuration, and distribution of vegetation can negatively impact the quality of the stream. The primary benefit of employing these methods lies in their cost-effective assessment, coupled with their capability to facilitate environmental monitoring and support informed decision-making for waterbody management.

However, several limitations were encountered. The resolution of the satellite image used was limited to 10 m, which is the best resolution achievable with freely available images. Higher resolution could have provided increased accuracy and more detailed detection of problem areas. Additionally, analyzing images captured during different seasons of the year could have contributed to a more comprehensive understanding of the vegetation structure. The season in which field surveys are conducted can also introduce bias due to variations in vegetation growth, water levels, and species composition. For instance, surveys during dry seasons may reveal more of the streambed, while wet seasons can difficult the identification of variations in the site-specific vegetation. To mitigate bias, assessments should ideally be conducted multiple times throughout the year to capture seasonal fluctuations accurately.

Moreover, the QBR index, which facilitates field-based assessment of riverine vegetation, permits subjective interpretations by researchers, potentially introducing two sources of error: method assumptions and observer bias. The first can be mitigated through observer training, as in this study, where final scores were assigned only after three observations, flora identification, and confirmation in the laboratory. The second arises when multiple observers are involved, requiring statistical analysis to minimize bias. Regarding the RSQI and NDVI, while the first provides insights into the LULC coverage along the stream, the second discerns the vegetation robustness. In this study, however, as not many types of vegetation were identified in the riparian area, both indices had very similar results and high correlation (r = 0.921, p < 00.05). Although the complementarity of the indices is relevant, perhaps this aspect would be better highlighted in studies covering a larger and more diverse vegetation area.

This study also has limitations regarding the scale selected for analysis. For future studies, analysis with scales at the basin level can offer a clearer view of the possibilities for reconnection of stream corridors and improvement of riparian areas.

### 4.4 Implications

The Banpo Stream, as a representative example of Seoul's streams, exhibits characteristics common to other streams, such as the Hongje, Dorim, Banghak, and Yeoui streams. These include disconnection from woodland areas due to road construction, a preference for concrete walking trails over vegetation introduction, and the introduction of species primarily for landscaping purposes. These streams also face limitations, such as narrow cross-sections and reduced channels, which restrict the availability of riparian vegetation. Moreover, the limited space available is often allocated to cycling paths and walking tracks, further reducing the area for water infiltration and vegetation growth. Consequently, by evidencing the negative impact of artificial structures in riparian areas on the ecological quality of urban streams, the findings of this study provide insights for similar cases and encourage the adoption of adaptive management measures.

Finally, this study makes contributions at various scales. At the local scale, it focuses on the restoration and assessment of the Banpo Stream in Seoul and provides recommendations for improving its ecological quality. The findings and recommendations are also relevant at the regional scale, as the study recognizes the common challenges faced by urban streams in Seoul and offers insights applicable to similar cases in the region. The interdisciplinary approach emphasized here promotes collaboration and a holistic understanding of stream restoration, which can inform research, policy development, and practical implementation not only in Seoul, but also in other urban areas globally, urging long-term monitoring and reassessment of measures.

To enhance future research, collaboration among students and professionals from diverse fields is essential to reduce knowledge gaps. Collecting information at different temporal and spatial scales is also crucial to gaining a better understanding of the dynamics of stream ecosystems and their relationships with the surrounding landscape.

## 5 Conclusion

The Banpo Stream, similar to many urban streams worldwide, faces significant ecological challenges caused by human activities and landscape alterations. The lack of natural riparian arrangements and extensive impervious surfaces contribute to reduced water quality, habitat fragmentation, and biodiversity loss. Additionally, the introduction of non-native species and the prioritization of recreational and aesthetic elements over ecological integrity in the riparian strip further deteriorate the water quality. This study underscores the importance of adopting the adaptive management approach in urban stream restoration, emphasizing continual reassessment and adjustment of restoration strategies based on monitoring data and stakeholder engagement. By integrating remote sensing techniques (RSQI and NDVI) and field observations (QBRI), this study provides valuable insights into the ecological conditions of the Banpo Stream and offers practical guidelines for improving urban stream restoration practices. Moreover, collaboration among multidisciplinary teams and long-term monitoring efforts are essential to address the complex challenges facing urban streams and promote sustainable management practices that balance human needs with ecological considerations.

## Funding

This research received no external funding.

## Data availability statement

The data that support the findings of this study are available from the corresponding author upon reasonable request.

## CRediT authorship contribution statement

**Jessica Tavares Machado:** Writing – original draft. **Gunwoo Kim:** Supervision.

## Declaration of competing interest

The authors declare that there is no potential conflict of interest.
